# Length of the Neurogenic Period—A Key Determinant for the Generation of Upper-Layer Neurons During Neocortex Development and Evolution

**DOI:** 10.3389/fcell.2021.676911

**Published:** 2021-05-13

**Authors:** Barbara K. Stepien, Samir Vaid, Wieland B. Huttner

**Affiliations:** ^1^Max Planck Institute of Molecular Cell Biology and Genetics, Max Planck Society (MPG), Munich, Germany; ^2^Institute of Anatomy, Faculty of Medicine Carl Gustav Carus, School of Medicine, Technische Universität Dresden, Dresden, Germany

**Keywords:** neocortex, evolution, neurogenic period length, gestation, upper-layer neurons

## Abstract

The neocortex, a six-layer neuronal brain structure that arose during the evolution of, and is unique to, mammals, is the seat of higher order brain functions responsible for human cognitive abilities. Despite its recent evolutionary origin, it shows a striking variability in size and folding complexity even among closely related mammalian species. In most mammals, cortical neurogenesis occurs prenatally, and its length correlates with the length of gestation. The evolutionary expansion of the neocortex, notably in human, is associated with an increase in the number of neurons, particularly within its upper layers. Various mechanisms have been proposed and investigated to explain the evolutionary enlargement of the human neocortex, focussing in particular on changes pertaining to neural progenitor types and their division modes, driven in part by the emergence of human-specific genes with novel functions. These led to an amplification of the progenitor pool size, which affects the rate and timing of neuron production. In addition, in early theoretical studies, another mechanism of neocortex expansion was proposed—the lengthening of the neurogenic period. A critical role of neurogenic period length in determining neocortical neuron number was subsequently supported by mathematical modeling studies. Recently, we have provided experimental evidence in rodents directly supporting the mechanism of extending neurogenesis to specifically increase the number of upper-layer cortical neurons. Moreover, our study examined the relationship between cortical neurogenesis and gestation, linking the extension of the neurogenic period to the maternal environment. As the exact nature of factors promoting neurogenic period prolongation, as well as the generalization of this mechanism for evolutionary distinct lineages, remain elusive, the directions for future studies are outlined and discussed.

## Introduction

The neocortex is the largest structure in the mammalian brain, covering most of its surface and being responsible for a substantial part of its computing capacity (Van Essen et al., 2018). The neocortex, which is characterized by six neuronal layers, has arisen relatively late in the evolution of vertebrates and is specific to mammals (Striedter, 2005). It is not only involved in basic sensory and motor processing but also enables higher cognitive functions, particularly in humans, where it underlies intellectual abilities such as language and abstract thinking. Major defects in the development of the neocortex can lead to severe cognitive impairments (Toi et al., 2009; Guarnieri et al., 2018).

Among mammals the neocortex shows a striking variability in size and degree of surface folding, often even between closely related species (Kelava et al., 2013; Zilles et al., 2013; Lewitus et al., 2014; Sun and Hevner, 2014; Namba et al., 2019). Neocortical surface folding is a mechanism that facilitates a pronounced increase in the surface area in relation to the overall size of the brain, allowing for more neural cells to be packed in the limited volume of the skull (Kelava et al., 2013; Zilles et al., 2013). Mammals can be broadly divided into two classes based on their neocortical morphology. Lissencephalic mammals, such as mouse and most other rodents, develop smooth neocortices lacking surface folds. In contrast, gyrencephalic species, to which humans belong, possess folded neocortices with multiple gyri and sulci. Even within the gyrencephalic species the degree of folding is highly variable and can be described by the gyrencephaly index (GI), which is typically calculated as the ratio of total surface area, including that of the sulci, to the exposed surface area (Elias and Schwartz, 1969; Zilles et al., 1988, 1989).

Although smaller mammals tend to have less folded neocortices, with the GI close to 1.0, the relationship between overall brain size and folding is not universal, with examples of large mammals such as the manatee having a lissencephalic cortex (Welker, 1990; Pillay and Manger, 2007; Lewitus et al., 2014; Vaid et al., 2018). Similarly, phylogenetic lineage relationships are also not determinative of cortex folding as exemplified by marmosets, which are lissencephalic, in contrast to most other primates, which possess gyrencephalic neocortices (Zilles et al., 1989; Kelava et al., 2012; Lewitus et al., 2014; Mitchell and Leopold, 2015; Vaid et al., 2018; Namba et al., 2019). Humans, next to cetaceans and elephants, are among the species with the highest GI (Zilles et al., 1988, 1989; Kelava et al., 2012; Lewitus et al., 2014; Namba et al., 2019). Humans also have the most expanded neocortex size in relation to body size compared to any mammal, especially in the frontal regions (Brodmann, 1912; Donahue et al., 2018). The underlying mechanisms and functional significance of such a disproportional size increase in terms of the evolution of human intelligence have been a subject of multiple studies and an intense scientific debate (Martin, 1996; Semendeferi and Damasio, 2000; Gibson, 2002; Semendeferi et al., 2002; Sherwood et al., 2005; Rilling, 2006; Azevedo et al., 2009; Herculano-Houzel, 2009; Barton and Venditti, 2013a, b; Montgomery, 2013; Sherwood and Smaers, 2013; Hofman, 2014; Gabi et al., 2016).

The characteristic structure of the neocortex with its six cytoarchitecturally distinct layers forms during the process of cortical neurogenesis, which in most placental mammals is completed prenatally (Clancy et al., 2001; Lewitus et al., 2014), with the exception of certain species such as ferret, where it continues for a short time after birth (Jackson et al., 1989). The length of this neurogenic period differs widely among mammalian species, e.g., in mouse, a small-brained lissencephalic mammal, the neurogenic period lasts only about 9–10 days (from embryonic day E10.5 to E18–E19.5) (Stepien et al., 2020), while in human, a large-brained gyrencephalic species, it lasts for around 110 days (from gestation week 10–25) (Clancy et al., 2001; Lewitus et al., 2014). Understanding the process of cortical neurogenesis, the evolutionary differences in this developmental program and how they arise, is necessary to explain the diversity of neocortical morphology and neuron populations among various mammalian species, particularly in relation to humans. In this review we will give a brief overview of the neocortical development and its evolutionary variations. Next, we will review the experimental evidence demonstrating the crucial role of the length of the neurogenic period in neocortical expansion, and finally propose directions for future studies, which should further explore and mechanistically explain the role of this mechanism in evolution.

## Cortical Neurogenesis and Its Temporal Sequence

### How Neurons Are Born—Progenitors and Their Diversity

Together with the olfactory bulb, amygdala, hippocampus and the basal ganglia, the neocortex develops from the telencephalic vesicles (Haines and Mihailoff, 2018). The characteristic six-layer structure of the cerebral cortex arises during the period of neurogenesis, which in placental mammals is mostly prenatal. Microscopically, the gray matter of the neocortex consists of six distinct neuronal layers, which form in an inside-out fashion during development (Shimada and Langman, 1970; Rakic, 1974, 1988). In the mature neocortex these layers contain two major populations of neurons: cortical projection neurons, which are generated locally (Noctor et al., 2001, 2004; Haubensak et al., 2004), and interneurons, which migrate into the neocortex from the ganglionic eminences (Anderson et al., 1997; Lavdas et al., 1999; Wichterle et al., 1999; Parnavelas et al., 2000).

During embryonic development, after neural tube closure, the future neocortex originates from a thin single cell layer of symmetrically dividing neuroepithelial cells (Rakic, 1995a, b), which exhibit notable apical-basal cell polarity (Götz and Huttner, 2005). Upon the onset of neurogenesis, neuroepithelial cells transform into apical radial glia (aRG) (Levitt et al., 1981; Malatesta et al., 2000; Campbell and Götz, 2002; Götz and Huttner, 2005; Taverna et al., 2014). aRG divide to produce neurons either directly (direct neurogenesis) or indirectly, by generating the more neuronally committed basal progenitors (BPs) (Noctor et al., 2001, 2004; Haubensak et al., 2004; Miyata et al., 2004). The cell body of aRG resides in the apical-most germinal zone, called ventricular zone (VZ) due to its contact with the brain lateral ventricles, and—like the neuroepithelial cells they derive from—form a pseudostratified epithelial layer (Götz and Huttner, 2005). These cells maintain a direct contact with both the apical (ventricular) surface as well as the basal lamina (pial or meningeal side) via apical and basal processes, respectively (Götz and Huttner, 2005). This contact can be lost at the basal side at later developmental stages in some gyrencephalic species as the cortical plate becomes thicker (Nowakowski et al., 2016). As a result, the basal processes of bRG become the main scaffold guiding the migration of supragranular neurons. As these processes exhibit a fan-like spatial distribution (Reillo et al., 2011), this in turn leads to an increased tangential spread of these neurons (Richman et al., 1975). Within the VZ, the position of the aRG cell body depends on the phase of the cell cycle, with S-phase occurring in the basal region of the VZ and mitosis at the ventricle, due to the process of interkinetic nuclear migration, hence the pseudostratified appearance (Sauer and Walker, 1959; Taverna and Huttner, 2010).

**FIGURE 1:**
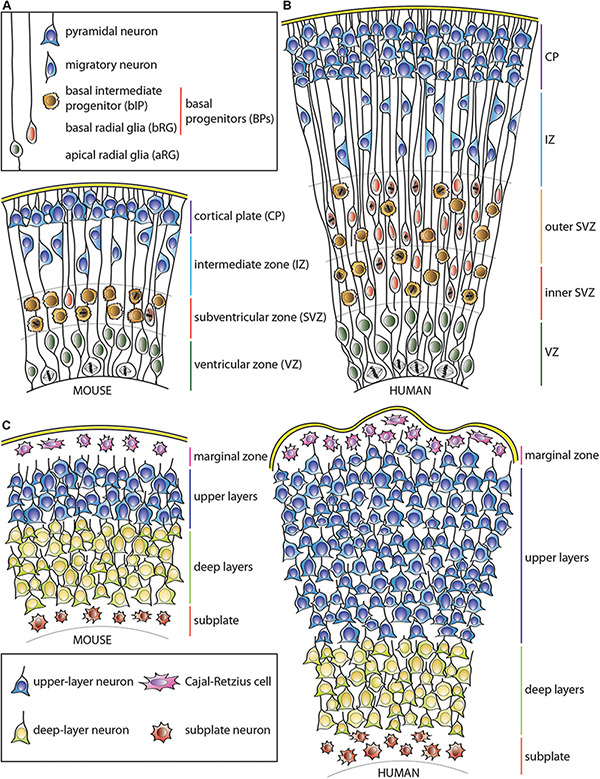
Schematic representation of the developing neocortical wall in mouse and human. **(A)** Mouse neocortical wall. **(B)** Human neocortical wall. **(C)** Mouse (left) and human (right) cortical plates. Note both the tangential and radial (predominantly in the upper layers) expansion of the human cortical plate. For cell types, see keys in **(A,C)**.

Dividing aRG can generate various types of BPs that lose their apical contact and migrate basally to the adjacent, more basal germinal layer—the subventricular zone (SVZ) (Haubensak et al., 2004; Miyata et al., 2004; Noctor et al., 2004; Gal et al., 2006; Fietz et al., 2010; Hansen et al., 2010; Stancik et al., 2010; Reillo et al., 2011; Betizeau et al., 2013; Pilz et al., 2013; Tyler and Haydar, 2013). The diversity of BP types, their proliferative capacity and relative abundance vary widely among mammalian species. In lissencephalic mammals, such as most rodents, including the mouse, most of the BPs have limited proliferative potential. Most abundant are basal intermediate progenitors (bIPs), which express the Tbr2 (Eomes) transcription factor (Englund et al., 2005). In the mouse, the overwhelming majority of bIPs divide symmetrically to produce two neurons, which explains their very limited proliferative potential (Haubensak et al., 2004; Miyata et al., 2004; Noctor et al., 2004). Additionally, there are also basal radial glia (bRG) progenitors, which were originally identified by the presence of a basal process (Fietz et al., 2010; Hansen et al., 2010; Reillo et al., 2011) and are now known to characteristically exhibit a radial morphology with apical and/or basal processes (Betizeau et al., 2013; Pilz et al., 2013). bRG have a greater proliferative potential than the canonical mouse bIPs as they can undergo symmetric proliferative and asymmetric self-renewing divisions (Fietz et al., 2010; Hansen et al., 2010; Reillo et al., 2011; Shitamukai et al., 2011; Wang et al., 2011; Betizeau et al., 2013; Pilz et al., 2013; Kalebic et al., 2019). These cells are, however, rare in the rodent SVZ (Wang et al., 2011), except for the medial neocortex (Vaid et al., 2018). Accordingly, the SVZ of the mouse, and similar mammals with small and mostly smooth cortices, is relatively thin and has a low proliferative capacity compared to that of the VZ (Figure 1A).

In contrast, in gyrencephalic mammals, particularly those with expanded and highly folded neocortices such as human, the complexity of the SVZ as well as the number of various BP cell types and their abundance are widely increased (Figure 1B; Fietz et al., 2010; Hansen et al., 2010; Reillo et al., 2011; Betizeau et al., 2013; Pilz et al., 2013; Kalebic et al., 2019). This is particularly true for the most proliferative type of BP, the bRG, although human bIPs also show capacity for symmetric proliferative divisions (Hansen et al., 2010). This leads to secondary expansion of the SVZ, which in gyrencephalic species, including humans, can be subdivided into two distinct germinal subzones—inner SVZ (iSVZ) and outer SVZ (oSVZ) (Smart et al., 2002). The iSVZ is mostly similar to the SVZ of the small-brained lissencephalic animals such as most rodents and relatively constant in thickness throughout neurogenesis (Smart et al., 2002). In contrast, the oSVZ expands substantially during neurogenesis due to the presence of highly proliferative BPs (Smart et al., 2002; Fietz et al., 2010; Hansen et al., 2010; Reillo et al., 2011).

This expansion of the germinal zones allows for the substantial increase in the cell clone size originating from a single VZ-resident aRG (Noctor et al., 2001; Reillo et al., 2011), and leads to the lateral spread of the daughter neurons over a larger surface area in gyrencephalic, as opposed to lissencephalic, species (Reillo et al., 2011; Kalebic et al., 2018). As a result, clonal cortical columns in folded cortices have a more conical shape (with the tip of the cone at the ventricle) in comparison to typical columns in the mouse neocortex (Figure 1C; O’Rourke et al., 1992, 1995, 1997; Kornack and Rakic, 1995; Reid et al., 1997; Ware et al., 1999; Reillo et al., 2011; Gertz and Kriegstein, 2015). Nonetheless, an abundance of bRG does not necessarily imply a highly folded neocortex, as some mammals, such as marmoset, have relatively small and smooth cortices despite a high abundance of bRG progenitors (Garcia-Moreno et al., 2012; Kelava et al., 2012). Similarly, in ferret, unlike human, bRG have been reported to contribute substantially to the generation of astrocytes compared to neurons (Reillo et al., 2011; Martinez-Cerdeno et al., 2012). Therefore, additional factors seem to control the proliferative potential of bRG as well as the ultimate fate of their progeny across different mammals. As an example, human-specific adaptations such as changes in mitochondrial metabolism (Florio et al., 2015; Namba et al., 2020) or increased morphological complexity of bRG (Betizeau et al., 2013; Kalebic et al., 2019) have been linked to increased proliferation and neuron production as well as neocortical folding.

### How Layers Are Made—Neuronal Migration

The newly formed cortical neurons do not remain in the germinal zones but instead undergo a migratory process in which they typically move basally along radial glia fibers, passing through the intermediate zone (IZ), and populate the expanding cortical plate (CP) (Caviness, 1982; Sheppard and Pearlman, 1997; Figure 1C). The first-born pioneer neurons form a preplate, which is subsequently separated into two distinct parts by the incoming later-born neurons, which settle between its lower and upper layers. Thereby the preplate becomes populated by two separate cell groups: Cajal-Retzius cells and subplate neurons, which constitute the basal-most layer I (marginal zone) and subplate region, respectively (Molnar and Blakemore, 1995; Super et al., 1998; Olson, 2014; Molnar et al., 2020). These neurons form temporary layers and are removed by apoptosis after the cortical plate is formed. The remaining layers II–VI of the neocortex form with the deep (most apically located) layer VI neurons being born earliest and the upper (most basally located) layers II-III neurons being born last (Angevine and Sidman, 1961; Rakic, 1974, 1988; Polleux et al., 1997a; Takahashi et al., 1999; Bystron et al., 2008; Klingler et al., 2019).

The newly born neurons use both aRG and bRG cell processes as a scaffold to guide their migration toward the basal side (Noctor et al., 2001; Reillo et al., 2011), and the later born neurons pass through the layers of earlier born neurons, hence the younger cells reside more basally than the older ones (Angevine and Sidman, 1961; Rakic, 1974; Kornack and Rakic, 1995; Polleux et al., 1997a; Takahashi et al., 1999; Parnavelas, 2000; Klingler et al., 2019). This inside-out sequence of neuron generation and migration is critical for proper wiring of the neocortex and its functionality, as evidenced by the phenotypes caused by mutations in critical components of this system (D’Arcangelo et al., 1995, 1997; Sheldon et al., 1997; Trommsdorff et al., 1999; Hong et al., 2000; Howell et al., 2000; Kuo et al., 2005; Lammert and Howell, 2016; Ishii et al., 2016).

Cortical neurogenesis is concluded when the progenitor pool either gets depleted due to symmetric consumptive divisions, or undergoes a fate switch, when the production of glial cells—astrocytes and oligodendrocytes—is initiated (Barnabe-Heider et al., 2005; Kessaris et al., 2006; Miller and Gauthier, 2007; Costa et al., 2009; Hirabayashi and Gotoh, 2010; Rowitch and Kriegstein, 2010; Beattie and Hippenmeyer, 2017; Tiwari et al., 2018; Ohtsuka and Kageyama, 2019). The onset of the period of gliogenesis follows that of the neurogenic period, with these periods exhibiting various degrees of temporal overlap across species. In the mouse, these periods are almost completely temporally separated, whereas in humans they proceed in parallel for a longer period of time (Levitt et al., 1983).

There are also temporal differences in the timing and rate of neuron production during neurogenesis across different regions of the neocortex, with rostral regions completing neurogenesis earlier than the caudal ones (Rakic, 1974; Gardette et al., 1982; Smart and Smart, 1982; Bayer and Altman, 1990; Sanderson and Weller, 1990; Miyama et al., 1997; Polleux et al., 1997a). While neurogenesis is thought to start around the same time across the whole cortex, the difference in its termination between rostral and caudal poles can vary from less than a day in mouse to about 3 weeks in monkeys (Finlay and Uchiyama, 2015). This phenomenon is referred to as a rostro-caudal gradient of cortical neurogenesis and has implications for the regional differences in neuron numbers and densities, relative growth rates, and the evolution of the neocortex (Charvet, 2014). The implications of the temporal structure of cortical neurogenesis and neuron migration for the evolutionary expansion of the neocortex are discussed in detail in the following chapters.

## Human Neocortex Expansion

The human brain is disproportionally expanded in relation to body size, and the part of the brain that shows the most striking expansion is the neocortex (Allman, 1999; Weaver, 2005; Rilling, 2006; Barton and Venditti, 2013a). In comparison to our closest relatives—the other great apes—the human neocortex has grown in terms of overall volume, surface area, and the number of neurons (Semendeferi et al., 2001; Herculano-Houzel et al., 2007; Donahue et al., 2018). It contributes to about 80% of our brain mass, with about 1,600–2,000 cm^2^ of folded surface area, and an average 2.6 mm thickness of the gray matter (see below) (Azevedo et al., 2009; Glasser et al., 2016; Donahue et al., 2018; Van Essen et al., 2018). The human cerebral cortex has been estimated to contain anywhere from 16 to 21–26 billion neurons depending on the counting method, which constitutes nearly 20% of the total neuron number in the entire human brain, i.e., including the cerebellum (Pelvig et al., 2008; Azevedo et al., 2009; Van Essen et al., 2018). The thickness of the human neocortex varies by region, and is more pronounced in association areas, such as the prefrontal cortex, than in the primary sensory and motor areas (Donahue et al., 2018).

Furthermore, the expansion of the human neocortex is not evenly distributed across different regions and neuronal layers. The rostral cortical areas are substantially more enlarged, particularly the frontal and prefrontal regions, both in terms of the overall volume, surface area as well as the amount of white matter (Brodmann, 1912; Deacon, 1997; McBride et al., 1999; Semendeferi and Damasio, 2000; Semendeferi et al., 2002; Schoenemann et al., 2005; Rilling, 2006; Passingham and Smaers, 2014; Smaers et al., 2017; Donahue et al., 2018), which has been attributed to increased connectivity and not necessarily to increased neuron numbers (see below) (Schoenemann et al., 2005; Barton, 2006; Collins et al., 2010; Gabi et al., 2016; Smaers et al., 2017; Donahue et al., 2018). The disproportionate increase in size, particularly of the prefrontal cortex, in comparison to other primates such as chimpanzees, has caused speculation as to its relevance with regard to human cognitive abilities (Brodmann, 1912; Armstrong et al., 1991; Deacon, 1997; Smaers et al., 2017).

The thickness of the neocortical gray matter also increases with brain size such that the average thickness of the human neocortex is approximately three times larger than that of the mouse (Hofman, 1988; Zhang and Sejnowski, 2000). At the microscopic level, the supragranular layers II–III are disproportionally expanded and show a greater increase in the number of neurons in species with larger cortices than the deep neuronal layers (Marin-Padilla, 1992; Hutsler et al., 2005; Molnar et al., 2006). This has been linked to the production of these neurons occurring late during neurogenesis. The exponential growth of late-produced layers is thought to stem from the expansion of the SVZ, which becomes a secondary highly proliferative zone in big-brained species (Smart et al., 2002; Pollen et al., 2015; Nowakowski et al., 2016). It also correlates with the prolongation of both neurogenic period and gestation length (Hutsler et al., 2005; Lewitus et al., 2014). An increase in the number of upper-layer neurons is more pronounced in the caudal as compared to rostral areas of the neocortex; this also agrees with the temporal rostro-caudal gradient of cortical neurogenesis, which is particularly stark in animals with larger cortices (Rakic, 1974, 2002; Gardette et al., 1982; Smart and Smart, 1982; Bayer and Altman, 1990; Sanderson and Weller, 1990; Miyama et al., 1997; Polleux et al., 1997a; Collins et al., 2010; Collins, 2011; Cahalane et al., 2012; Charvet, 2014; Charvet and Finlay, 2014; Charvet et al., 2015).

## Neurogenic Period Length in Brain Expansion—Theoretical Concepts

**FIGURE 2:**
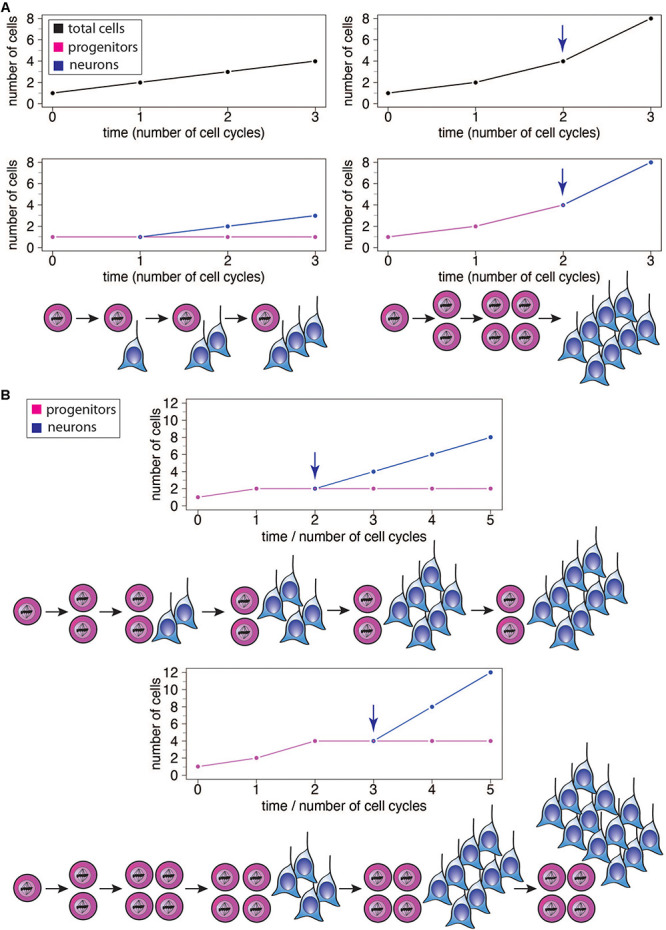
**(A)** Illustration of the effects of different modes of progenitor division on the number of neurons produced, showing the results after 3 cell cycles each. Left: Asymmetric neurogenic progenitor divisions (1 progenitor: >1 progenitor + 1 neuron; see diagram) lead to a linear increase in total cell number with every cell cycle (top graph, black), with the number of progenitors remaining constant (bottom graph, magenta) and the number of neurons increasing linearly after the first cell cycle (bottom graph, blue). Right: In contrast, symmetric divisions lead to an exponential increase in total cell number with every cell cycle (top graph). The initial symmetric proliferative progenitor divisions (1 progenitor: >2 progenitors; see diagram) double the number of progenitors with every cell cycle (bottom graph, magenta). Upon progenitors switching to symmetric consumptive neurogenic division (arrow), the number of neurons (bottom graph, blue) is twice that of the (now consumed) progenitors. The longer the neurogenic period and the greater the number of progenitor cell cycles, the greater the increase in the number of neurons generated in the right scenario compared to the left one. **(B)** Illustration of the effects of delaying the switch of symmetric proliferative progenitor divisions to asymmetric neurogenic progenitor divisions (see top and bottom diagrams). Progenitors undergo one (top) or two (bottom) cycle(s) of symmetric proliferative division and then switch to asymmetric neurogenic progenitor divisions, with the number of progenitors then generated remaining constant and the number of neurons increasing linearly. The number of neurons produced upon each asymmetric neurogenic progenitor division matches the number of progenitors present. Because the number of progenitors is twice as high after two (bottom) than one (top) cycle(s) of symmetric proliferative division, the number of neurons added upon each cycle of asymmetric neurogenic progenitor division is twice as high in the bottom than top scenario, leading to a steeper increase in neuron numbers and a greater neuron output in the bottom scenario, although neurogenesis starts one progenitor cycle later.

The increase in the absolute size of the human brain, and particularly the neocortex, is thought to largely reflect an increase in the proliferative capacity of the relevant progenitor cells and adaptive changes in the temporal structure of neural development (Rakic, 2002). An increase in progenitor number by symmetric proliferative divisions introduces an intrinsic exponential component (Figure 2A right). As a result, prolongation of such a proliferative phase has a disproportionally larger impact on the structures that arise later in development. Accordingly, brain structures such as the neocortex, or its specific regions that appear late in development, tend to disproportionally increase in size relative to early developing ones (Finlay and Darlington, 1995; Finlay et al., 1998; Rakic, 2002). In this scenario a simple extension of the length of neurogenesis, during which at least a fraction of progenitor divisions is symmetric proliferative, would result in an expansion of the brain structure concerned by progressively increasing the rate of progenitor production and hence the numbers of neurons generated therefrom (Rakic, 1988, 2002; Figure 2A right). It is worth noting here that it is also possible for an extension of the neurogenic period to cause a linear increase in the final number of neurons generated, without progressively increasing the rate of neuron production, if one assumes that the additional progenitor divisions resulting from that extension would be exclusively asymmetric neurogenic (Figure 2A left). The exact magnitude of the effect of neurogenic period prolongation is therefore dependent on the specific types of progenitors present in a given species and their mode of division. In species which possess a highly proliferative progenitor pool, even a modest extension of neurogenesis would lead to a dramatic increase in the rate of neuron production over time, and a far greater final neuronal output (Figure 2A right), while in species with primarily asymmetric neurogenic progenitor divisions, the addition of neurons per unit time would be largely constant and the final rise in neuron number modest (Figure 2A left). In this context, it is important to point out that even for an—in principle—same progenitor-to-neuron lineage, for example with progenitors first undergoing symmetric proliferative divisions and then asymmetric self-renewing neurogenic divisions, the same number of progenitor cell cycles will result in a greater neuron output if progenitors undergo symmetric proliferative divisions for one cycle more and, accordingly, asymmetric self-renewing neurogenic divisions for one cycle less (Figure 2B).

An increase in the length of the neurogenic period has therefore been explored as an attractive mechanism to explain the relative size difference in a given brain structure between various species. The analysis of over 100 mammals showed that the changes in the relative size of non-olfactory brain structures as compared to the total brain largely occur in a non-linear way, and the largest size increases are observed for the late-developed brain structures (Finlay and Darlington, 1995). Thus, the enlargement of a given brain structure in relation to the increase in total brain size due to prolonged neurogenesis has exponential and linear components, depending on the division mode of the various progenitors at specific timepoints (Finlay and Darlington, 1995; Finlay et al., 2001). The temporal structure of neurogenesis, which is largely conserved across mammalian species (Finlay and Darlington, 1995; Darlington et al., 1999; Clancy et al., 2001), would determine the extent of this expansion, with late structures expanded more relative to overall brain size (Finlay and Darlington, 1995).

**FIGURE 3:**
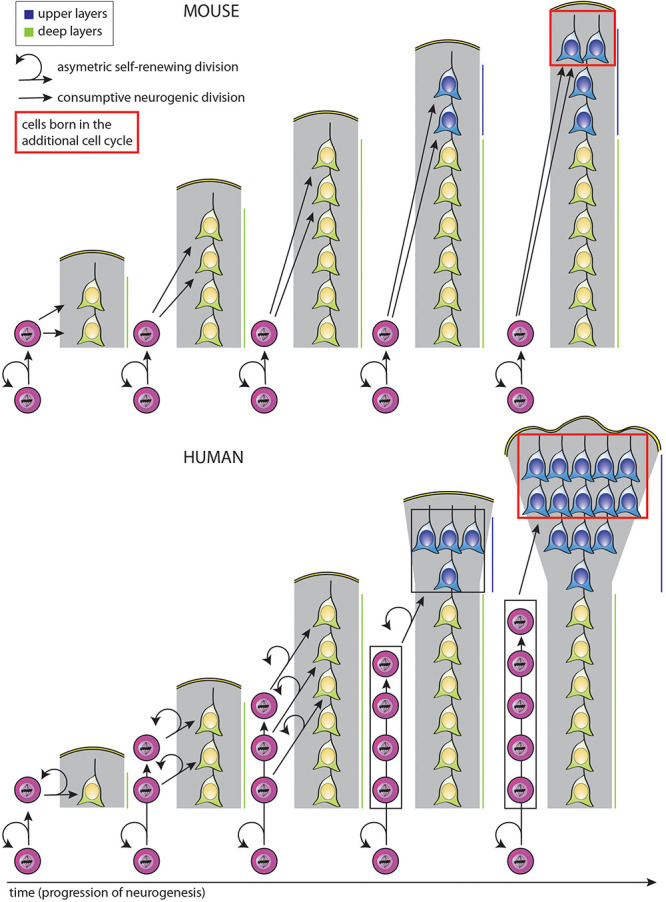
Simplified model illustrating how a selective increase in upper-layer neurons in human as compared to mouse can be achieved by changing the type of BP generated from aRG and the mode of BP division, with the same temporal progression regarding the change from deep-layer to upper-layer neuron generation. In both mouse (top) and human (bottom), aRG (bottom cells in each diagram) undergo repeated cycles of asymmetric, self-renewing and BP-genic, division. However, the type of BP generated and its mode of division over the course of neurogenesis are different. Top: With each cycle of asymmetric self-renewing division (round arrows), an aRG in embryonic mouse neocortex generates one bIP (straight vertically pointing arrows), which then undergoes consumptive neurogenic division, generating two neurons (pairs of straight oblique arrows). This leads to a linear increase in the total number of neurons generated, with two neurons added per single aRG division cycle. Bottom: With each cycle of asymmetric self-renewing division (round arrows), an aRG in fetal human neocortex generates one bRG (straight vertically pointing arrows). This undergoes repeated cycles of asymmetric, self-renewing division (round arrows), with the other daughter being a neuron (straight oblique arrows). As each bRG persists due to self-renewal, this leads to a linear increase in the number of bRG with each aRG cycle. This in turn leads to a progressive increase in the number of neurons generated per aRG cycle, which is equal to that in mouse after the 3rd aRG cycle, that is, an equal number of deep-layer neurons has then been generated in both the mouse and human scenario. However, if we assume that for both mouse and human, BPs switch to generate upper-layer neurons in the 4th aRG cycle, due to the accumulation of bRG in human, a greater number of upper-layer neurons in generated in this cycle in human than mouse. This difference becomes even greater if in the next cycle, the bRG in human, like the bIPs in mouse, adopt a consumptive neurogenic mode of division (red boxes), perhaps via the generation of bIPs (not illustrated; for a more detailed depiction of possible BP lineages, see Lewitus et al., 2014). *Comments*: (i) Hence, lengthening the neurogenic period, e.g., from the 4th to the 5th aRG cycle, results in a selective increase in upper-layer neurons for both mouse and human; (ii) a lineage of asymmetric self-renewing BP-genic aRG division followed by asymmetric self-renewing neurogenic bRG division followed by consumptive neurogenic bRG division (see above) results in a greater upper-layer neuron output in human than the lineage of asymmetric self-renewing BP-genic aRG division followed by consumptive neurogenic bIP division in mouse.

Importantly, the selective expansion of the upper neuronal layers of the neocortex, a hallmark of neocortex expansion (Marin-Padilla, 1992; Hutsler et al., 2005; Molnar et al., 2006), could also be the result of a similar mechanism, in that the additional progenitor divisions at the end of a longer neurogenic period specifically generate excess supragranular neurons (Marin-Padilla, 1992; Hutsler et al., 2005; Molnar et al., 2006; Charvet et al., 2015; Figure 3, see comment in legend). Moreover, the temporal progression regarding the change in the type of neuron generated over the course of neurogenesis, i.e., from deep-layer to upper-layer neurons, could be the same even when the particular progenitor lineage and modes of progenitor division are distinct, e.g., for mouse vs. human (Figure 3; McConnell and Kaznowski, 1991; Frantz and McConnell, 1996; Desai and McConnell, 2000; Haubensak et al., 2004; Shen et al., 2006; Gaspard et al., 2008; Kowalczyk et al., 2009). In the hypothetical scenario illustrated in Figure 3, changes in the type of BP generated from aRG and in the mode of BP division alone suffice to explain a selective increase in upper-layer neuron generation in human as compared to mouse, with no difference between mouse and human in the number of deep-layer neurons generated in spite of the differences in BP type and mode of division.

To summarize, the final number of cortical neurons produced is determined (i) on the one hand by the various types of progenitor cells, their pool sizes and lineages, and their division modes over the course of neurogenesis, and (ii) on the other hand by the overall length of this process. The first-mentioned set of progenitor features has been shown to be different across various mammalian species and to undergo evolutionary changes at the genomic level (Florio et al., 2017; Namba and Huttner, 2017; Cardenas and Borrell, 2020; Vaid and Huttner, 2020). Differences in these progenitor features between species could be sufficient to explain the differences in neuron production. However, whether in species with the same progenitor features changes in the length of the neurogenic period could by itself also suffice to explain the differences in neuron production is less clear. Moreover, if this were the case, it would be important to determine whether progenitor-intrinsic or extrinsic factors cause the lengthening of the neurogenic period. In the following sections, we discuss the neurogenic period prolongation hypothesis as a cause for neocortical expansion, in both mathematical modeling as well as experimental studies.

## Mathematical Models of Cortical Neurogenesis

As cortical neurogenesis is a complex process, it can only be understood by simultaneously taking into account the spatiotemporal changes in the behavior of the various cell types of this tissue. Several mathematical modeling approaches have been used to describe and make testable predictions about cortical neurogenesis both for given model species and for comparing evolutionarily distinct mammals.

Early models of mouse cortical neurogenesis concentrated primarily on the two main parameters: cell cycle kinetics, and the temporal changes in the proportions of differentiative vs. proliferative progenitor divisions (Takahashi et al., 1996, 1997; Polleux et al., 1997b; Nowakowski et al., 2002). One such model explored the relationship between the length of the neurogenic period, expressed as a number of progenitor cell cycles, and the fraction of cell cycle exits in time, to predict the final number of neurons generated (Takahashi et al., 1997). Although this model was based on a simplified description of progenitor cell behavior and a shorter than real length of the neurogenic period, the authors were able to show that both the length of neurogenesis and the fraction of cell cycle exits could be manipulated *in silico* to achieve larger or smaller cortices. Importantly, of these two parameters, the prolongation of neurogenesis had a drastically larger effect on the final neuronal output (Takahashi et al., 1997).

The recent accumulation of observational and experimental data pertaining to mammalian cortical neurogenesis, as well as to other developmental, physiological and ecological traits, extending beyond a few model species, has triggered renewed attempts to model the process of neurogenesis in evolution *in silico*. These mathematical models have been aimed in particular at elucidating the developmental mechanisms behind two main outcomes of the evolutionary variation of cortical neurogenesis: the differences between the numbers of cortical neurons generated, and the change in the GI across various mammalian species (Gohlke et al., 2007; Cahalane et al., 2014; Lewitus et al., 2014; Mota and Herculano-Houzel, 2015; Picco et al., 2018).

The study by Picco et al. (2018) used simplified parameters accounting for proliferative, asymmetric and terminal neurogenic divisions of progenitors to analyze neurogenesis in three mammalian species: mouse, macaque and human. This model postulated the critical importance of the timing of the switch from the dominance of proliferative and asymmetric neurogenic to terminal neurogenic divisions in determining the final neuronal output. Interestingly, the effect of differences in the initial progenitor pool size was small, which led to the unexpected—and factually incorrect—prediction of a lower initial progenitor pool for human than for macaque. Although this model offered a minimal framework for assessing the effects of the balance between different progenitor division modes on neuron production, its broader biological significance is unclear.

Another modeling study has attempted to explain the evolutionary increase across species in the number of neocortical neurons and the concomitant spatial patterns of their distribution (Cahalane et al., 2014). Using existing data on the cell cycle length and exit kinetics, as well as apoptosis rate, derived from 15 mammalian species, the authors showed that changes in these parameters allow for a massive variation in cortical neuron numbers, with minimal contribution from the variations in initial progenitor pool size. The model also predicted (i) the known regional differences in neuron distribution, namely the increased neuron number in posterior cortical regions, consistent with the rostro-caudal gradient of neurogenesis (Rakic, 1974, 2002; Gardette et al., 1982; Smart and Smart, 1982; McSherry and Smart, 1986; Bayer and Altman, 1990; Sanderson and Weller, 1990; Caviness et al., 1995; Miyama et al., 1997; Polleux et al., 1997a; Nowakowski et al., 2002; Collins et al., 2010; Collins, 2011; Cahalane et al., 2012; Charvet, 2014; Charvet et al., 2015), and (ii) the thickening of the upper cortical layers, which reflects a disproportionately increased number of late-born neurons (Marin-Padilla, 1992; Hutsler et al., 2005; Molnar et al., 2006; Charvet et al., 2015). Additionally, in agreement with empirical data (Rakic, 1974; McSherry and Smart, 1986; Collins et al., 2010; Cahalane et al., 2012; Charvet, 2014; Charvet and Finlay, 2014; Charvet et al., 2015), these differences were found to be more pronounced in species with larger cortices (Cahalane et al., 2014). Although manipulating the above-mentioned basic parameters was sufficient to accurately predict final neuron number and distribution, the model offered little explanatory insight as to how a fine tuning of developmental events, e.g., by changing the proportion of different progenitor pools, could contribute to driving cortical expansion.

A more comprehensive mathematical modeling study (Lewitus et al., 2014) aimed at explaining the variation of GI and cortical neuron numbers across mammals using known neuronal progenitor lineages and their characteristics. The authors examined over a 100 mammalian species with respect to their neocortical size and folding as well as a number of physiological and life history traits. These species were found to segregate into two distinct groups separated by a threshold GI value of about 1.5. This separation depended on the existence of a subpopulation of symmetrically dividing proliferative BPs in the SVZ of highly gyrencephalic mammals and predicted different rates of neuron production. Among species in either group, when accounting for the initial progenitor pool size and a given combination of progenitor lineages leading to neurons, the final number of cortical projection neurons produced depended on a single parameter—the length of the neurogenic period (Lewitus et al., 2014). This finding implies that within each of the two groups of mammals identified based on their GI, the differences in the number of cortical neurons produced can be explained by shortening or prolonging the time of neurogenesis, without changes in progenitor lineages or modes of progenitor division. In fact, in accordance with experimental data (Herculano-Houzel, 2009), this model predicts the human cerebral cortex to be just a scaled-up version of the cerebral cortex of other primates, in which the increased neuron number can be explained by lengthening of the neurogenic period by about 8 days. The length of neurogenesis, a process that in most mammals occurs primarily prenatally, was also shown to strongly correlate with the average length of gestation (Lewitus et al., 2014).

Taken together, the hypothesis that cortical neurogenesis length is a decisive parameter in determining neuronal output, particularly among closely related species with similar progenitor populations, emerged from a number of mathematical modeling studies (Takahashi et al., 1997; Cahalane et al., 2014; Lewitus et al., 2014; Picco et al., 2018). In the next section, we present available experimental evidence supporting this hypothesis, and explore the nature of potential determinants of neurogenesis length.

## Role of Neurogenic Period Length in Neocortex Expansion—Experimental Evidence

### Genes Affecting Cortical Progenitor Division and Neurogenic Period Length

Although neurogenic period length has emerged in theoretical and modeling studies as a crucial parameter determining neuron numbers during the evolutionary expansion of the neocortex, addressing the role of neurogenic period length directly by experimental approaches has been challenging. This has been mostly due to a lack of tools allowing for the specific manipulation of neurogenic period length independent of affecting other parameters of cortical neurogenesis. Instead, most studies addressing neocortex expansion during evolution have aimed at identifying and characterizing genomic differences between small- and large-brained species, typically humans and rodents (Namba and Huttner, 2017; Cardenas and Borrell, 2020; Vaid and Huttner, 2020), and recently also apes (Prescott et al., 2015; Mora-Bermudez et al., 2016; Otani et al., 2016; Rosales-Reynoso et al., 2018), that lead to an increase in neocortical neuron production, with its resulting consequences. The latter include thickening of the cortical wall, particularly of its upper layers, and the tangential expansion of the neocortex, resulting in increased surface folding (Florio et al., 2015, 2018; Ju et al., 2016; Liu et al., 2017; Namba and Huttner, 2017; Fiddes et al., 2018; Kalebic et al., 2018; Suzuki et al., 2018; Cardenas and Borrell, 2020; Heide et al., 2020; Vaid and Huttner, 2020). Although a number of evolutionarily changed genes have been shown to regulate the primary progenitor pool by affecting the division mode of aRG (Fish et al., 2006; Buchman et al., 2010; Nicholas et al., 2010; Gruber et al., 2011; Pinson et al., 2019), changes across species in the genome that affect the proliferative potential of BPs are of particular interest (Florio et al., 2015, 2018; Ju et al., 2016; Fiddes et al., 2018; Suzuki et al., 2018; Heide et al., 2020; Vaid and Huttner, 2020), given the expansion of the oSVZ in the human lineage (Fietz et al., 2010; Hansen et al., 2010; Lamonica et al., 2012; Betizeau et al., 2013; Pfeiffer et al., 2016). Indeed, the increased BP proliferation induced by overexpression of such genes, e.g., of the human-specific genes *ARHGAP11B* or *NOTCH2NL* (Notch 2 N-terminal like), in model species was found to induce features commonly associated with neocortex expansion, such as a specific increase in upper-layer neurons or cortical folding (Florio et al., 2015; Ju et al., 2016; Liu et al., 2017; Kalebic et al., 2018; Heide et al., 2020). Of note, forced expression of human-specific *ARHGAP11B* in developing ferret neocortex was found to prolong neurogenesis (Kalebic et al., 2018), as is discussed further below. The challenge is to determine whether any effect on the length of the neurogenic period that is associated with the increased BP proliferation is the result of the latter, or an independent effect (for example, delaying the fate switch of progenitors from producing neurons to producing glia, or postponing the acquisition of quiescence of progenitors) caused by such genomic changes.

### *In vitro* Models of Cortical Neurogenesis

Some of the more informative studies addressing this question include comparative studies that examine the evolutionary changes in the dynamics of cortical neurogenesis *in vitro*, as the culture systems allow for easier tracking of developmental events over a wide time window with frequent sampling. The basic program of cortical neurogenesis, including the formation of fluid-filled ventricle-like cavities, progression through specific progenitor cell lineages, and sequential generation of deep- and upper-layer neurons, can be recapitulated by neural precursors, derived from embryonic stem cells or induced pluripotent stem cells *in vitro*, in both 2D and 3D cultures (Eiraku et al., 2008; Gaspard et al., 2008; Shi et al., 2012; Lancaster et al., 2013; Mora-Bermudez et al., 2016; Otani et al., 2016). Such culture systems have been used to compare the behavior of neural progenitor cells derived from rodent and primate species (Eiraku et al., 2008; Gaspard et al., 2008; Espuny-Camacho et al., 2013; Mora-Bermudez et al., 2016). These studies show that both mouse and human cells follow an intrinsic developmental program; however, the sequential steps of neurogenesis were found to be protracted in the human culture, leading to generation of larger structures (Eiraku et al., 2008; Lancaster et al., 2013; Espuny-Camacho et al., 2013). Mouse progenitors generated the first neurons around 7 days after the initiation of neuronal differentiation in the *in vitro* cultures, and completed neuron production within approximately 20 days after initiation of differentiation, while human cells started neurogenesis around 2–4 weeks after initiation of differentiation and continued producing neurons for much longer, up to 15 weeks (Gaspard et al., 2008; Espuny-Camacho et al., 2013).

Following these findings, a comparative study characterized the temporal dynamics of the progenitor cell populations in macaque, chimpanzee and human using 2D and 3D *in vitro* cultures, uncovering major differences in their behavior (Otani et al., 2016). In contrast to mouse BPs, which mostly generate neurons and hence do not exhibit significant self-amplification (Noctor et al., 2001, 2004; Haubensak et al., 2004), in the above-mentioned primates progenitor self-amplification occurs in parallel with neurogenesis, consistent with *in vivo* findings (Otani et al., 2016). This leads to a substantially larger neuronal output of primate, compared to rodent, progenitor cells. Importantly, the authors also demonstrated profound differences in the timing of neurogenesis among primate species (Otani et al., 2016). Human progenitors prolonged their proliferative phase compared to macaque progenitor cells. Thus, while human progenitors kept expanding their pool size exponentially up to 20 days longer than macaque cells, macaque progenitors switched to asymmetric division earlier, which led to a linear rather than exponential increase in the size of their progeny. In addition, macaque progenitors generated a substantial proportion of progeny that exited the cell cycle earlier than human progenitors. Furthermore, chimpanzee and human progenitors switched from producing deep-layer neurons to upper-layer neurons later than macaque progenitors. These species-specific temporal features were cell-autonomous, as they remained unaffected by the co-culture with progenitors of another species.

Taken together, these *in vitro* findings point to the existence of intrinsic temporal differences in neurogenic events among different mammalian species. First, there are species-specific temporal differences in cell production rate (Otani et al., 2016), which likely depend on the division mode of the relevant progenitor type. Second, the *in vitro* systems at least partly recapitulate the overall lengthening of neurogenesis in human compared to mouse and, to a lesser extent, macaque. These findings could explain the differences in the final number of neurons produced in culture; however, they do not fully account for the *in vivo* situation. Importantly, as described above, few significant differences between human and chimpanzee neurogenesis timing *in vitro* have been reported (Otani et al., 2016), despite an over twofold difference in the number of cortical neurons between these two species *in vivo* (Herculano-Houzel et al., 2007). The differences in the timing of neurogenesis between human and non-human primates observed *in vivo* vary from those observed *in vitro*, which may reflect certain limitations of the latter model systems, in particular regarding the development of an oSVZ. This issue has recently been competently discussed (Betizeau and Dehay, 2016). This discrepancy could be resolved by the conclusions from the mathematical modeling study (Lewitus et al., 2014), which suggests that for species with similar progenitor types and lineages, such as human and chimpanzee, the bulk of the difference in neuron production can be explained by simply prolonging the neurogenic period by a few days. Such subtle changes in neurogenesis length may not be easily recapitulated *in vitro*. Interestingly, timing differences at earlier stages of cortical development modeled *in vitro* between human and other great apes have been noted in a recent study (Benito-Kwiecinski et al., 2021). In this system, neuroepithelial cells in human cerebral organoids delayed the switch to a more mature transition morphotype prior to the onset of neurogenesis compared with chimpanzee and gorilla. This led to a shorter cell cycle length of human neuroepithelial cells and an increase in the number of neurogenic progenitors leading to more neurons.

### Links Between Progenitor Behavior and Length of Neurogenesis

Another unsolved question is whether the changes in progenitor proliferative potential, and hence in the different rates of cell production, and the differences in the length of the neurogenic period across species are causally connected. A recent *in vivo* study with relevance to this question points to a potential link. Specifically, in exploring the effects of the human-specific gene *ARHGAP11B* on neocortex expansion, both an increase in the proliferation of BPs and a prolongation of the neurogenic phase was observed when that gene was overexpressed in the developing ferret neocortex, resulting in greater neuron production (Kalebic et al., 2018). Moreover, *ARHGAP11B* expression led to a specific increase of upper-layer neurons, recapitulating a hallmark of the evolutionary expansion of the neocortex. In spite of these observations, it remains unclear by what mechanism increasing the proliferative capacity of progenitors could be linked to the prolongation of neurogenesis. Similarly, it remains to be determined if increasing the proliferative capacity of progenitors somehow affects the timing of deep-layer vs. upper-layer neuron production, or whether these two phenomena are mechanistically distinct. Hence, the challenge has been to obtain experimental evidence either for or against a causal link between the proliferative capacity of progenitors and the length of the neurogenic period.

### *In vivo* Evidence for a Role of Neurogenic Period Lengthening in Neocortex Expansion

We recently addressed the potentially critical role of a prolongation of the neurogenic period on increased neocortical neuron production. In a nutshell, our study directly demonstrates that lengthening of the neurogenic period can induce hallmarks of neocortex expansion, without overt alterations in the existing progenitor lineages (Stepien et al., 2020). Our study used a mouse model system to explore a possible causative relationship between the prolongation of the neurogenic period and the increased number of neocortical neurons produced, without introducing genetic changes that would affect the lineage of progenitors. Given the correlative evidence linking the length of neurogenesis with gestation length (Lewitus et al., 2014; Glatzle et al., 2017), we took advantage of a number of inbred mouse strains, previously characterized to have a genetically determined difference of about 1–2 days in the average gestation length. This corresponds to about 5–10% of the total length of mouse pregnancy (Murray et al., 2010). We could show that the strains with a longer gestation produced significantly more cortical neurons than the short-gestation strains (Stepien et al., 2020). Moreover, this increase in the number of neurons was brought about by extending the neurogenic period by about 1 day, and—accordingly—pertained specifically to the late-produced upper-layer neurons of the neocortex.

Moreover, there were no major alterations to the rate of neuron production between short- and long-gestation strains (Stepien et al., 2020). That is consistent with a steady rate of neurogenesis in mice due to the dominance of asymmetric neurogenic aRG divisions (Miyata et al., 2001; Noctor et al., 2001; Haubensak et al., 2004). Therefore, the increase in upper-layer neuron production was due to a longer retention of a pool of cycling neurogenic progenitors, rather than an increased rate of neuron production per unit time, in the long-gestation mice (Stepien et al., 2020). In the latter scenario, there would be an increase in neuron generation rates throughout neurogenesis (Polleux et al., 1997b), which was not observed. That neuronal add-on to the existing neurogenic program occurred without altering the timing of the fate switch between deep-and upper-layer neuron production, which took place at around embryonic day 14.5 (Stepien et al., 2020), as reported previously (Saurat et al., 2013; Toma et al., 2014; Klingler et al., 2019).

We also addressed potential mechanisms leading to the prolongation of the neurogenic period (Stepien et al., 2020). Given the correlation of upper-layer neuron production and neurogenesis length with the length of gestation, which has been found to be maternally determined in the mouse strains analyzed (Murray et al., 2010) as well as in other mammals (Zhang et al., 2017), we hypothesized that the maternal environment could provide cues controlling cortical neurogenesis timing. To test this hypothesis, the embryos of the short-gestation strain were transferred into long-gestation strain foster mothers, and *vice versa* (Stepien et al., 2020). The short-gestation strain fetuses that developed in the maternal environment of the long-gestation strain fosters were found to specifically increase the number of upper-layer, but not deep-layer, cortical neurons generated, to the level characteristic of the foster mother phenotype. Conversely, long-gestation strain fetuses developing in short-gestation strain mothers showed a diminished number of upper-layer neurons generated, in accordance with the maternal phenotype. These results show that, at least in mouse, maternal factors are sufficient to drive the prolongation of neurogenesis in a non-cell autonomous way, independent of the genotype and local environment of the embryo.

Although our study—to the best of our knowledge—is the first direct demonstration of the causative effect of the lengthening of the neurogenic period on the expansion of upper-layer cortical neurons (Stepien et al., 2020), such a role in interspecies evolution remains unclear. One piece of evidence pointing to the conservation of such a role between different mammalian species was obtained by analyzing a rat-mouse chimeric embryo generated in our study. The chimeric embryo, generated by injecting mouse ES cell into a rat morula, was allowed to develop in a rat foster mother, and its neocortex was then analyzed at E19.5 to compare the number of cortical neurons produced to that of plain mouse or rat embryos. Interestingly, while the number of deep-layer neurons was comparable between embryos of either species and the chimeric embryo, the number of upper-layer neurons in the chimeric neocortex was greater than that of a mouse, but comparable to a rat, embryonic neocortex, regardless of whether these neurons were generated by mouse or rat progenitors (Stepien et al., 2020). This finding is consistent with a key role of the maternal environment in controlling the production of cortical neurons, likely by its effect on the length of neurogenesis.

## Future Directions—Factors Controlling Neurogenic Period Length

### Progenitor-Intrinsic vs. Extrinsic Signals

Lengthening of the neurogenic period has emerged as a so far little studied mechanism driving the evolutionary expansion of the neocortex. Existing experimental data suggest that an increase in the neurogenic period length could result from either a change in the intrinsic properties of progenitor cells, an alteration of cell-extrinsic signals, possibly derived from the maternal environment, or from a combination of these two sources. Thus far the exact contribution of cell-intrinsic and -extrinsic factors is elusive, with some seemingly contradictory findings.

While our study of neurogenic period lengthening in mice has shown that cell-extrinsic, maternally-controlled factors can control this process (Stepien et al., 2020), comparative studies of human, apes and ferret *in vitro* and *in vivo* (Otani et al., 2016; Kalebic et al., 2018) suggest genetically encoded cell-intrinsic properties of progenitors can also be responsible. If these findings were regarded as a discrepancy, there could be various explanations. First, in our study of mouse inbred strains, by concentrating on the *intra*species differences, we were able to separate the effect of neurogenic period length from other possible evolutionary changes in progenitor lineages and their proliferative capacity (Stepien et al., 2020). Although the latter differences between inbred mouse strains cannot be completely excluded, their effect on the observed phenotype is negligible as evidenced by the results of the embryo transfer experiments. In contrast, comparing *inter*species differences necessarily includes the confounding effects of a multitude of genetic changes affecting progenitor biology. These clearly would alter the rate of neuron production in the course of neurogenesis, due to changing the balance between symmetric proliferative, asymmetric neurogenic and terminal progenitor divisions.

**FIGURE 4:**
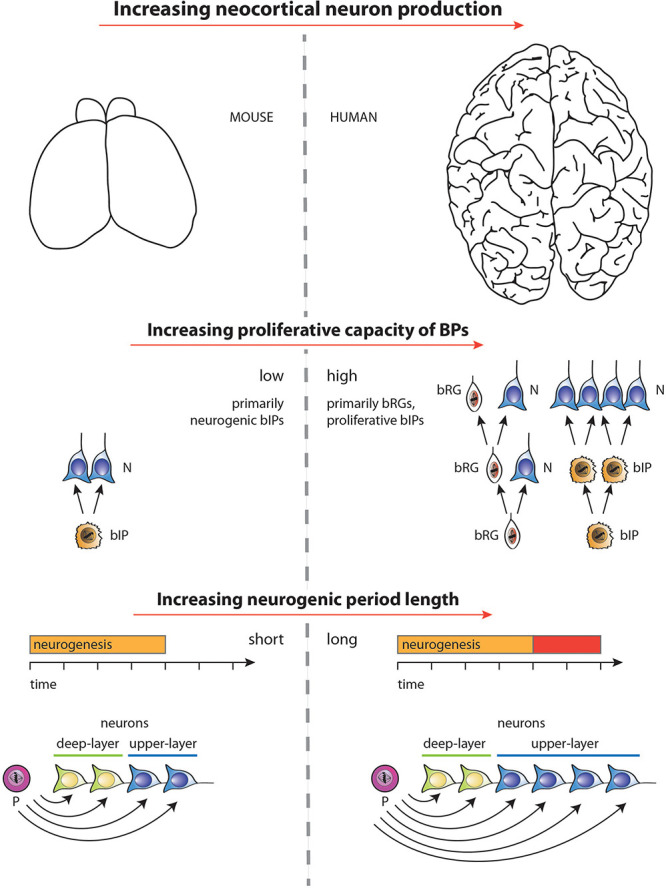
Diagram illustrating the two major factors underlying the increase, during development, in neuron production associated with the evolutionary expansion of the neocortex, as depicted for mouse vs. human in the top row (images not drawn to scale). (1) Middle row: Increase in the proliferative capacity of BPs by changing the type of BPs and their mode of division. BPs in embryonic mouse neocortex comprise mostly bIPs of which each one undergoes a single consumptive division generating two neurons (N). BPs in fetal human neocortex comprise both bRG and bIPs, both of which can undergo various modes of cell division (see Lewitus et al., 2014) of which the following are illustrated. bRG may undergo repeated asymmetric self-renewing divisions generating one neuron each. bIPs may first undergo symmetric proliferative divisions, resulting in an exponential increase in their number, followed by consumptive neurogenic divisions of these bIPs. (2) Bottom row: Selective increase in upper-layer cortical neurons upon lengthening the neurogenic period. Once progenitors (P) that generate neurons (N) have switched from generating deep-layer neurons to generating upper-layer neurons, a lengthening of the neurogenic period (red bar) during neocortex development, e.g., in fetal human as compared to embryonic mouse, will result in selectively increasing upper-layer neurons. For simplicity, and to illustrate the underlying principle, irrespective of the actual lineages in embryonic mouse vs. fetal human neocortex (see middle row), in the example illustrated, a progenitor is assumed to successively generate first deep-layer and then upper-layer neurons by repeated asymmetric self-renewing divisions, which occur for longer in fetal human than embryonic mouse neocortex. Not illustrated—for the ease of presentation—are other lineage scenarios, in which with a mixed population of progenitors, some progenitors undergo symmetric proliferative divisions while others undergo neurogenic, e.g., symmetric consumptive, divisions, with both types of progenitor divisions occurring for longer in species developing an expanded neocortex.

In this context, it should be noted that increasing the intrinsic proliferative potential of BPs can also lead to changes in the timing of various neurogenic events, such as delaying the switch from deep-layer to upper-layer neuron production, and increasing the overall length of neurogenesis (Kalebic et al., 2018). Whether the effects on the progenitor division mode can be uncoupled from the temporal events, or if they are linked by an underlying biological mechanism, is an open question. Nonetheless, it is increasingly likely that the lengthening of the neurogenic period, i.e., between human and closely related primate species, requires two independent components—one linked tightly to evolutionary changes in the proliferative potential of neural progenitors, the other—uncoupled from that process—controlled by cell-extrinsic, potentially maternally-derived factors. Figure 4 summarizes, for embryonic mouse and fetal human neocortex, the two major factors underlying the increase in neuron production associated with the evolutionary expansion of the neocortex— (i) increasing the proliferative capacity of BPs, and (ii) increasing the length of the neurogenic period.

### Toward Identifying Extrinsic Signals

While the role of cell-intrinsic, particularly human-specific, factors controlling progenitor proliferative capacity have been the subject of numerous studies (Namba and Huttner, 2017; Cardenas and Borrell, 2020; Vaid and Huttner, 2020), the nature of cell-extrinsic factors, including those potentially derived from the maternal environment, is unknown. A role of the maternal environment in the evolution of brain and neocortex size has been hypothesized previously, based on correlative evidence (Pagel and Harvey, 1988; Martin, 1996; Lewitus et al., 2014). One of the early hypotheses suggested the maternal basal metabolic rate and energy expenditure as a rate-limiting factor in prenatal brain growth (Martin, 1996). This model explained the link between maternal metabolic rate, offspring’s brain size, and gestation length by suggesting that for a given brain size the decrease in the metabolic rate of the mother results in simultaneous extension of the gestation period in order to provide sufficient energy for neurogenesis. However, the underlying assumptions of this model have since been questioned (Barton, 2006). Thus, the bulk of neocortical neurogenesis is completed in most species during the prenatal period, whereas the timing of neurogenesis in relation to birth can be widely different (Clancy et al., 2001; Workman et al., 2013).

The limiting factor for studying the effects of maternal environment on neocortical development, particularly in relation to the evolutionary lengthening of both gestation and neurogenic period, is the complexity of the interaction between maternal and fetal compartments. Studies of factors determining the physiological gestation length are few and far between, although most point to a primarily maternal nature of these factors, involving multiple genetic loci (Murray et al., 2010; Zhang et al., 2017; Ewert et al., 2018; Rodrigues et al., 2020). In light of the multiplicity of genetic loci involved, a genetic manipulation of gestation period length that goes beyond small intraspecies variation appears to be challenging, if not impossible, in the near future. Studying chimeric animals, as shown in our study (Stepien et al., 2020), offers an approach to manipulate the maternal environment of the developing brain; however, this approach is currently limited to closely related species with similar gestation lengths, as obtaining animals with high degree of chimerism from more divergent species has so far been unsuccessful (Wu et al., 2017; De Los Angeles et al., 2018; Fu et al., 2020).

### Maternally-Derived Extrinsic Signals

An alternative approach entails searching directly for potential molecular factors that could be transferred from the maternal environment into the developing fetal nervous system. A number of biologically active substances are already known to affect brain development due to pathologies caused by their dysregulation. Here, small molecule metabolites, nutrients and hormones such as thyroid hormones (Stepien and Huttner, 2019; Batistuzzo and Ribeiro, 2020), retinoic acid (McCaffery et al., 2003), folic acid (Ramaekers et al., 2013; Balashova et al., 2018), and gut flora-derived compounds (Vuong et al., 2020) have received particular attention. It would be interesting to test if evolutionary changes in the production, transport and local metabolism of these compounds can affect the timing of neurogenesis. Such a role has been proposed for thyroid hormones (Stenzel and Huttner, 2013; Stepien and Huttner, 2019), in light of their known role in controlling heterochrony in other contexts (Faunes and Larrain, 2016) and of the fact that thyroid hormone signaling components show profound differences in expression and functionality between mammalian species (Stepien and Huttner, 2019). Interestingly, binding of thyroid hormones and activation of integrin αvβ3 was shown to increase BP proliferation (Stenzel et al., 2014). This particular integrin is also upregulated in the human oSVZ compared to the mouse SVZ (Fietz et al., 2012), pointing to a potential evolutionary mechanism for BP amplification.

To affect neurogenic period length directly, potential maternal factors must by necessity be able to act over a long range. To enter the developing brain, they have to pass through multiple barriers, most crucially the placenta and the developing blood-brain barrier (Goasdoue et al., 2017; Landers and Richard, 2017; Saunders et al., 2019; Stepien and Huttner, 2019). When considering potential delivery routes to the developing neocortex, two compartments are particularly relevant: the neurovasculature and the cerebrospinal fluid (CSF) system. A future detailed characterization of the chemical composition of blood and CSF and its changes in both ontology and evolution is likely to result in the identification of candidate compounds, which can subsequently be tested for their role in controlling cortical neurogenesis and its length. This approach, although laborious and requiring adequate bioinformatic analysis of complex datasets, would be unbiased and would allow the identification of candidate molecules, irrespective of whether they are transferred from maternal to fetal compartment directly, or are produced downstream in response to maternal signals. Hopefully, the rising interest of the scientific community in the CSF and neurovascular systems (Fame and Lehtinen, 2020) may well lead to breakthrough discoveries concerning such factors.

## Conclusion

There is an accumulating body of evidence that the increase in the length of the neurogenic period is a crucial factor underlying neocortical expansion during evolution. The fundamental role of this determinant, initially predicted over 30 years ago (Rakic, 1988, 2002), has been supported by both mathematical modeling studies and, more recently, experimental data. Nonetheless, the robust determination of the importance of neurogenic period length in the increase of human brain size, and mammalian brain evolution in general, has been hampered for various reasons. These include (i) the lack of reliable information on the length of the neurogenic period in a sufficiently large collection of species, (ii) the possible interplay between progenitor proliferative capacity and neurogenic period length *per se* in determining neuronal output, and (iii) the lack of suitable model systems that can easily be manipulated. Our finding that various inbred mouse strains differ in the length of cortical neurogenesis, which in turn results in changes in the final number of neurons generated, opens up avenues for further mechanistic studies. In addition, the identification of the maternal environment as a determinative factor in neurogenic period prolongation offers a direction for the elucidation of the molecular players that control such prolongation. Together with a characterization of neurogenesis in an increasing number of mammalian species, an elucidation of genetic and cell biological differences in progenitor behavior, and a refining of *in vitro* model systems such as brain organoid cultures, the various findings discussed in this review should provide a basis for a better understanding of the evolutionary changes underlying the development of the human neocortex.

## Author Contributions

BS, SV, and WH made substantial contributions to the conception of this work. BS wrote the original draft. SV and WH revised it critically. WH approved the final version of this manuscript. All authors contributed to the article and approved the submitted version.

## Conflict of Interest

The authors declare that the research was conducted in the absence of any commercial or financial relationships that could be construed as a potential conflict of interest.
